# Relationship and sexual satisfaction among patients with bipolar disorder and partners

**DOI:** 10.1093/sexmed/qfaf051

**Published:** 2025-07-22

**Authors:** Lærke Helene Andreasen, Vita Djamilla Sandeman, Helle B Krogh, Julie Ravneberg Stokholm, Caroline Fussing Bruun, Jeff Zarp, Lars Vedel Kessing, Annamaria Giraldi, Maj Vinberg

**Affiliations:** Sexological Clinic, Copenhagen University Hospital – Mental Health Services CPH, Ole Maaløes Vej 14, 2100 Copenhagen, Denmark; Mental Health Centre, Northern Zealand, Copenhagen University Hospital – Mental Health Services CPH, Dyrehavevej 48, 3400 Hillerødn, Denmark; Sexological Clinic, Copenhagen University Hospital – Mental Health Services CPH, Ole Maaløes Vej 14, 2100 Copenhagen, Denmark; Mental Health Centre, Northern Zealand, Copenhagen University Hospital – Mental Health Services CPH, Dyrehavevej 48, 3400 Hillerødn, Denmark; Sexological Clinic, Copenhagen University Hospital – Mental Health Services CPH, Ole Maaløes Vej 14, 2100 Copenhagen, Denmark; Copenhagen Affective Disorders Research Centre (CADIC), Psychiatric Center Copenhagen, Nordre Fasanvej 57, 2000 Frederiksberg, Denmark; Copenhagen Affective Disorders Research Centre (CADIC), Psychiatric Center Copenhagen, Nordre Fasanvej 57, 2000 Frederiksberg, Denmark; Copenhagen Affective Disorders Research Centre (CADIC), Psychiatric Center Copenhagen, Nordre Fasanvej 57, 2000 Frederiksberg, Denmark; Copenhagen Affective Disorders Research Centre (CADIC), Psychiatric Center Copenhagen, Nordre Fasanvej 57, 2000 Frederiksberg, Denmark; Copenhagen Affective Disorders Research Centre (CADIC), Psychiatric Center Copenhagen, Nordre Fasanvej 57, 2000 Frederiksberg, Denmark; Department of Clinical Medicine, Faculty of Health and Medical Sciences, University of Copenhagen, Blegdamsvej 3B, 2200 Copenhagen, Denmark; Sexological Clinic, Copenhagen University Hospital – Mental Health Services CPH, Ole Maaløes Vej 14, 2100 Copenhagen, Denmark; Department of Clinical Medicine, Faculty of Health and Medical Sciences, University of Copenhagen, Blegdamsvej 3B, 2200 Copenhagen, Denmark; Mental Health Centre, Northern Zealand, Copenhagen University Hospital – Mental Health Services CPH, Dyrehavevej 48, 3400 Hillerødn, Denmark; Department of Clinical Medicine, Faculty of Health and Medical Sciences, University of Copenhagen, Blegdamsvej 3B, 2200 Copenhagen, Denmark; The Early Multimodular Prevention and Intervention Research Institution (EMPIRI), Dyrehavevej 48, 3400 Hillerød, Denmark

**Keywords:** Bipolar disorder (BD) (mesh), relationship satisfaction, sexual satisfaction, sexual function, couples, Couple Satisfaction Index (CSI), spouses (mesh)

## Abstract

**Objectives:**

Good interpersonal relationships are associated with improved functioning, quality of life, and a better prognosis in patients with bipolar disorder (BD). Little information is available regarding relationship satisfaction and sexual satisfaction within couples where 1 partner has BD.

**Aim:**

This cross-sectional study aimed to examine relationship and sexual satisfaction in patients with BD and partners to patients with BD.

**Methods:**

Patients with BD and partners to patients with BD were included, and outcomes were assessed using semi-structured interviews and questionnaires.

**Outcomes:**

Couple satisfaction was measured by the self-reported questionnaire Couple Satisfaction Index (CSI-4), and sexual satisfaction was measured by 3 self-reported questions. Multiple regression analyses were used to compare the groups adjusting for sex, age, mood symptoms, overall functioning, and stress symptoms. The results were compared to general populations.

**Results:**

One hundred eleven patients with BD and 74 partners were included. We found a significant difference between patients with BD and partners concerning relationship satisfaction measured with the CSI, with partners being less satisfied (*P* = .050). Comparing relationship satisfaction in patients with BD and partners to the general population, we found that the general population was more satisfied in each CSI item (*P* < .050). In multiple regression analyses adjusted for sex, age, mood symptoms, stress, and function, patients with BD were more satisfied with their sexual life over the last year compared to partners (*P* = .039). They further rated the importance of a good sexual life higher than partners (*P* = .006). Finally, more patients with BD and partners rated their sex life the last year as being bad to extremely bad compared to the control group from the general population (partners = 21.1%, BD = 23.4%, general population = 16%).

**Clinical implications:**

In clinical practice, it is essential to focus on relationships including sexual life in patients with BD and partners as both groups have a lower degree of relationship and sexual satisfaction compared to the general population.

**Strengths and limitations:**

The use of validated questionnaires and clinical ratings is a strength, albeit the cross-sectional design is a limitation.

**Conclusions:**

Patients with BD reported a higher degree of satisfaction with their relationship and sexual life compared to their partners. Compared to the general population, both groups expressed lower degree of relationship and sexual satisfaction.

## Introduction

Marriage or living with a partner positively affects physical and mental health and is associated with a longer life span.^1^ Social support has a protective effect on patients’ well-being, especially when coming from a partner.^2^ For most people, sexual life is an important part of a relationship^3^ and impacts the quality of life.^4^ Sexual life includes sexual activity, relationships, and intimacy. Sexual function is understood through a biopsychosocial approach, which acknowledges that biological, psychological, interpersonal, and sociocultural factors all influence sexual function^5^^,^^6^ and interact over time.^5^^,^^6^

Bipolar disorder (BD) can be a severe psychiatric disorder. For the majority, it has its onset in adolescence, and it is characterized by interchanges between euthymic, (hypo)manic, manic, mixed, and depressed episodes.^7^ Patients with BD are more likely to live alone compared to the general population^8^ and are more frequently divorced or separated from their partners.^8^ Furthermore, patients with BD have a decreased lifespan of 8-12 years compared to the general population.^9^^,^^10^ Patients with BD, who are in a relationship, have fewer subclinical symptoms during periods of remission^8^ and better medical compliance.^11^ Therefore, emphasizing the examination of relationships holds clinical significance in BD.

Changes in sexual desire are linked to BD as increased sexual interest can be a symptom of mania and decreased sexual interest is a classic characteristic of depression.^7^ Only a few studies have investigated the relationship between individuals with BD and partners’ sexual and relationship satisfaction. It is reasonable to assume that alignment in libido and expectations of one’s sexual life plays a vital role in ensuring couples’ satisfaction.^12^ Indeed, previous studies have shown that both patients with BD and their partners reported higher levels of marital^8^^,^^13^ and sexual dissatisfaction.^12^^,^^13^ Still, there is consensus that more research is needed in the area.^14^^,^^15^ Thus, having BD can have an impact on both patients with BD and their partners’ sexual life and relationship satisfaction.

Having a mental health illness increases the risk of sexual dysfunction,^16^ with sexual dysfunction defined as a sexual problem that causes distress.^17^ Like other mental health illnesses, there is a higher prevalence of sexual dysfunction among patients with BD compared to the general population^18^ even in euthymic periods.^18^ Additionally, as psychotropic medicine has multiple adverse sexual effects, medical treatment for BD may result in sexual dysfunction.^10^

Being in a relationship with an individual with a severe physical or mental disorder will often lead to increased caregiver burden. Caregiver burden refers to physical, emotional, financial, or social challenges that caregivers may experience due to providing care and support, which impact their well-being and quality of life.^19^ When the patient with BD is experiencing a manic or depressive episode, the partner experiences more domestic responsibilities.^20^ Partners of patients with BD receive less emotional support than those in a relationship with individuals without BD,^11^ and the partners’ engagement in the treatment can be energy-consuming.^21^ They also experience more mental distress, even more than first-degree relatives,^19^ and a negative impact on their self-esteem and confidence.^21^

Knowing the comprehensive consequences for partners to patients with BD, it is also essential to focus on the partners’ mental health.^19-21^ A good and supportive relationship is significant for one’s health, possibly even more so for people with chronic illnesses such as patients with BD.^8^^,^^11^ As sexual life is essential for maintaining a satisfying relationship and has an impact on quality of life, the research on sexual and relationship satisfaction is crucial in understanding mental health, as both are closely interrelated and serve as significant indicators of overall well-being. We therefore aimed to study relationship and sexual satisfaction among patients with BD and their partners and to compare the results to the general population.^22^ We aimed to investigate the satisfaction among patients with BD and partners regarding sexual life and relationship and hypothesized that both groups would be less satisfied compared to controls, leading to the following research questions:

“*How is relationship and sexual satisfaction among patients with BD and partners to patients with BD?*”

“*Are partners and patients with BD less satisfied with their relationship and sexual life than the general population?*”

## Methods and materials

### Study samples

Patients with BD and partners were recruited from a Mood Disorder Clinic, a specialized clinic that provides treatment services for patients with newly diagnosed BD,^23^ who have been diagnosed within a maximum of 2 years. The participants were not offered compensation.

#### Patients with BD

*BIDISEX* is an observational study examining sexuality in patients with BD using clinical interviews, ratings, and questionnaires. The present part included data from inclusion of patients with newly diagnosed BD in a randomized controlled trial (RCT), the A-Bipolar RCT.^24^ Recruitment started in January 2022 and ended in February 2024. In total, 111 patients with BD were included. Inclusion criteria: age ≥ 18 years, a diagnosis of BD according to ICD-10^7^ confirmed by Schedules for Clinical Assessment in Neuropsychiatry (SCAN-interviews).^25^

#### Partners

*The R-bipolar* ^26^ is an RCT parallel-group trial consisting of partners participating in group-based psychoeducation for relatives of patients with BD.^26^ Recruitment started in April 2022 and was completed in January 2024. In total, 74 partners to patients with BD were included. Inclusion criteria: age ≥ 18 years, and partners to an individual with BD. It must be noted that partners included in this study were not necessarily partners to the patients with BD included in this study.

To compare relationship satisfaction in patients with BD and partners to a general population, and as there is no Danish validation study on this scale, we identified a recent validation study examining the Couple Satisfaction Index 4 (CSI-4) based on a sample from Canada.^27^ The median age of the Canadian population was 45 years, and all participants were married. The Canadian population shares key demographic and cultural similarities with Danes, and the recent validation ensures robust, up-to-date data for establishing reliable norm scores.

Furthermore, to compare sexual satisfaction, we used data from a large prospective Danish cohort study including over 75 000 citizens between 15 and 89 years of age.^3^^,^^28^ As the largest age group for both patients with BD and partners was between 25 and 34 years, we compared patients with BD and partners to the same age group in the general population on the sexual measures. Of further relevance 50% of these controls had a partner at the time of participating.

### Ethics

All participants signed informed consent before entering the study. *The R-bipolar study*^26^ was approved by Centre for Data Protection (P-2021–809) and allowed to be initiated without permission from the Scientific Ethical Committees of the Capital Region (case number 21063013). *The A-Bipolar Study* was approved by the Danish Research Ethics Committee (H-21014515) and the Data Agency, Capital Region of Copenhagen (P-2021-576).^29^

*The BIDISEX study* ^30^ was further approved by both the Centre for Data Protection (P-2021-809) and the Ethics Committee (case number 21063013).

### Instruments

#### Clinical ratings

##### Hamilton Depression Rating Scale

To assess depressive symptoms in the last 3 days, we used the clinical rating *Hamilton Depression Rating Scale (HDRS-17).*^31^ The HDRS-17-total score was categorized as *13-17 = mild depression, 18-24 = moderate, and 25-52 = severe.* HDRS-17 item 14 was moderated and used as a sexual item (Table 1) but not excluded from the total scores.

**Table 1:** Overview of items measuring relationship satisfaction and sexual satisfaction.

Domain	Questionnaire/rating	Questions/item	Answer options	Modified answer options
**Relationship satisfaction**	CSI4	Item 1: Please indicate the degree of happiness, all things considered, of your relationship.	Item 1: 0-6	
		Item 2: I have a warm and comfortable relationship with my partner.	Item 2: 0-5	
		Item 3: How rewarding is your relationship with your partner?	Item 3: 0-6	
		Item 4: In general, how satisfied are you with your relationship?	Item 4: 0-6	
Sexual satisfaction	SEXUS	LYST03: “Thinking about the last year, how would you characterize your own and your spouse’s/partner’s desire to have sex?”	LYST03: 1. Less sexual desire than partner 2. Equal desire for sex 3. Higher sexual desire than partner	
		SQOL01: “How important is having a good sex life to you? By sex life, we mean both masturbation and having sex with another person.”	SQOL01: 1. Not at all important 2. Not very important 3. Important 4. Very important 5. Extremely important	SQOL01: 1. Not at all/not very important 2. Important 3. Very/extremely important
		SQOL02: “Overall, how would you rate your sex life in the last year?”	1. Very bad 2. Bad 3. Neither good 4. Good **5**. Very good.	SQOL02: 1. Extremely/bad 2. Neither good nor bad 3. Extremely/good
**Sexual satisfaction**	HDRS-17	Item 14: Genital symptoms (symptoms such as loss of libido) Reduced sexual interest. Normal/no reduced sexual interest	0. Absent 1. Mild 2. Severe	0. No disturbances present 1. Disturbances present
**Sexual satisfaction**	YMRS	Item 3: Increased sexual interest.	0. Normal: not increased 1. Mildly or possibly increased 2. Definite subjective increase on questioning 3. Spontaneous sexual content; elaborates on sexual matters; hypersexual by self-report 5. Overt sexual acts (toward patients, staff, or interviewer)	0. No disturbances present 1. Disturbances present
**Sexual satisfaction**	FAST	Item E21: “Having satisfactory sexual relationships”	0. No difficulty 1. Mild difficulty 2. Moderate difficulty 3. Severe difficulty	0. Satisfying sexual relations 1. Not satisfying sexual relations

Couple Satisfaction index 4 (CSI4), Hamilton depression rating scale (HDRS-17), Young Mania Rating Scale (YMRS), Functioning assessment short test (FAST).

##### Young Mania Rating Scale

To assess (hypo)manic symptoms in the last 3 days, the clinical rating instrument *Young Mania.*

*Rating Scale (YMRS)* ^32^ was used. YMRS scores are categorized as *16-22 = mild mania, 23-31 = moderate mania, and 32-60 = severe mania.* YMRS item 3 was moderated and used as a sexual item (Table 1) but not excluded from the total scores.

##### Functioning Assessment Short Test

To assess the everyday functioning level in daily life over the last 14 days, the observer-based *Functioning Assessment Short Test* (FAST)^33^ was used. FAST total score ranges from 0 to 72, where higher scores indicate a lower level of functioning, and is categorized as *no (0-11), mild,*^11-19^  *moderate,*^20-39^ and *severe (>40).*^33^ FAST item E21 was moderated and used as a sexual item (Table 1) but not excluded from the total scores.

#### Questionnaires

##### Couple Satisfaction Index

The *Couple Satisfaction Index 4 (CSI-4)* was used to measure relationship satisfaction.^34^ The CSI-4 is a self-report questionnaire consisting of 4 items (Table 1)*.* The score is between 0 and 23. Higher scores indicate higher relationship satisfaction. Scores below 13.5 indicate notable relationship dissatisfaction.^34^ A validation study of the scale showed a Cronbach’s alpha of 0.95 and a McDonald’s omega of 0.96.^27^

Only 12 recipients of partners to patients with BD answered all 4 items. Therefore, a total score consisting of the first 3 items was used and referred to as CSI-3-total for both partners and patients with BD.

##### Sexual satisfaction questions

Three questions (please see Table 1) from the project *SEXUS* were used.^3^^,^^28^ Project SEXUS is a Danish cohort study that has used a self-administered online questionnaire that was developed and validated in Danish.^35^ The question regarding sexual satisfaction in the last year (SQOL02) was the main outcome when assessing sexual satisfaction.

##### Perceived Stress Scale

The Perceived Stress Scale (PSS)^36^ is a self-reported questionnaire assessing participants’ perceived stress levels over the last 4 weeks. It consists of 10 questions where a higher score indicates a higher level of stress: *low (0-13), moderate,*^13-25^ and *high.*^26-39^

##### Short Form 12

Partners to patients with BD answered the questionnaire *Short Form 12 (SF12),*^37^ to assess perceived mental and physical health in the last 4 weeks.

## Statistical analyses

Independent *t*-tests were used to evaluate differences in clinical variables between the 2 groups, patients with BD and partners, and Pearson’s chi-square test was used to examine differences in categorical demographic and clinical variables. Continuous data were presented as median and interquartile range, while non-parametric and categorical data were presented as numbers and percentages.

Spearman’s correlation analysis was used to analyze bivariate correlations between CSI-3, the 3 SEXUS questions, total scores from HDRS-17, YMRS, FAST, and PSS, and the sexual items: HDRS-17 item 14, YMRS item 3, and FAST item 21.

A priori, it was decided to investigate the possible influences of covariates age, sex, HDRS-17, and YMRS total scores, as well as FAST total score, and perceived stress (PSS total score). Multiple regression analyses, including these covariates, which are known to influence sexual function and relationship satisfaction, were used. Our main outcomes were continuous data, including the CSI-3 total score representing couples’ satisfaction and SQOL02, representing sexual satisfaction and the categorical sexual items from HDRS-17, YMRS, and FAST scales, and the 3 SEXUS questions. The 3 SEXUS questions are presented in percentages for comparison reasons.^3^^,^^28^

Using multiple linear regression, we adjusted the outcome: CSI-3 total score, representing couples’ satisfaction, and the SQOL02, representing sexual satisfaction, in 3 regression models, adding the covariates that differed significantly between the patients with BD and partners. First, model 1 was adjusted for sex and age. Second, model 2 was adjusted for sex, age, HDRS-17, and YMRS scores.

Third, model 3 functioning (FAST) and stress (PSS) scores were added, and this was considered as the fully adjusted regression model. Partners were set as reference scores; thus, the *β*-coefficients represent the value for patients with BD. An adjusted *P*-value <.05 was considered statistically significant, and all analyses were conducted using the Statistical Package for Social Sciences (SPSS, version 29).^38^

Due to the use of electronic questionnaires, there were no missing values as participants could not submit the questionnaires without completing them.

## Results

### Demographic characteristics

In this study, 111 patients with BD and 74 partners were included. The demographic and clinical characteristics can be seen in Table 2. Comparing the distribution of sex, more partners than patients with BD were men (59.5 % vs. 48.6%). The partners were older (33.5) than patients with BD (33.0).

**Table 2:** Demographic characteristics, clinical ratings, and questionnaires for partners and patients with bipolar disorder (BD).

**Category**		**Partners**	**Patients with BD**
**Participants**		74	111
**Sex** (male) %		44.0 (59.5)	54.0 (48.6)
**Age (interquartile [25;75])**		33.5 [27;43.5]	30.0 [25;43]
18-34 years (%)		44.0 (59.4)	69.0 (62.2)
>35 years (%)		30.8 (40.6)	42.0 (37.8)
**Cohabitation**			
Yes		66.0 (89.2)	65.0 (58.6)
No		7.0 (9.4)	20.0 (18.0)
Other		1.0 (1.4)	26.0 (23.4)
**Years of education**			
Mean (SD)		16.12 (1.2)	15.1 (3.0)
**Employment (%)**			
Employed		54.0 (72.9)	44.0 (39.5)
Unemployed^a^		5 (6.8)	32 (28.8)
Students		15 (20.3)	35 (31.5)
Number of children			
Mean (SD)		0.86 (1.2)	0.69 (1.1)
0		41.0 (55.4)	74 (66.8)
>1		33.0 (44.6)	37.0 (33.3)
**Psychiatric diagnoses** ^**b**^ **(%)**			
At the moment yes^c^		12.0 (16.2)	
Previously		13.0 (17.6)	
Not diagnosed but thinks he/she has one		2.0 (2.7)	
No		47.0 (63.5)	
**Sex of partner (%)**			
Male		26.0 (35.1)	
Female		47.0 (63.5)	
Other		1.0 (1.4)	
**BD Type 1/2 (%)**			49%
**Age of onset (interquartile [25;75])**			18.0 [15;20]
**Medication, n (%)**			
	Lithium		53 (47.7)
	Antidepressive		5 (4.5)
	Antipsychotic		51 (45.9)
	Lamotrigine		58 (52.3)
	Use of benzodiazepines^d^		7 (6.4)^*^
**HDRS-17**			
Mean (SD)		5.0 (4.1)^*^	9.7 (6.3)
**Item 14**	*Reduced sexual interest*	17.0 (23.0)	39.0 (35.5)
	*Normal/no reduced sexual interest*	57.0 (77.0)	71.0 (64.5)
**YMRS**			
Mean (SD)		1.4 (1.8)^*^	5.2 (4.6)
Item 3	*Increased sexual interest*	5.0 (6.8)^*^	24.0 (21.8)
	*Normal/no increased sexual interest*	68.0 (93.2)	86.0 (78.2)
Mean (SD)		5.8 (5.5)^*^	18.0 (12.1)
Item E21	*Not satisfying sexual relation*	30 (40.5)	41 (37.3)
	*Satisfying sexual relations*	44 (59.5)	69 (62.7)
**SF-12**			
Mean (SD)		39.5 (11.7)	
Mean (SD)		53.6 (7.8)	
**PSS**			
Mean (SD)		18.4 (5.4)^*^	20.5 (6.9)
	*Low*	15.0 (20.3)	15.0 (13.5)
	*Moderate*	59.0 (79.7)	79.0 (71.2)
	*High*	0 (0.0)	17.0 (15.3)
**CSI-3**			
Mean (SD)		11.1 (3.3)	11.6 (3.3)
	*Item 1*	3.8 (1.3)	4.1 (1.4)
	*Item 2*	3.8 (1.1)	4.1 (1.1)
	*Item 3*	3.5 (1.2)	3.5 (1.1)

Continuous variables are presented as median [interquartile range]. Categorical variables are presented as number and %. Abbreviation: SD: standard deviation.

^*^A Statistically significant differences between partners and patients with BD

a Retired, military service, transfer income, sick leave, searching for a job, etc.

b Elaborated in ICD-10 diagnosis codes (International Classification of Diseases).

c Distribution in numbers of partners with previous and present psychiatric disorders, with it being possible to have more than 1 diagnosis: F30-F39 (only unipolar depression): n = 11.0, F40-F48: n = 12.0, F80-F89: n = 3.0, F90-98: n = 4.0.

d One patient received benzodiazepines daily, 6 per necessary.

HDRS-17, Hamilton Depression Rating Scale with 17 items version; YMRS, Young Mania Rating Scale; FAST, The Functional Assessment Short Test; WHOQOL, A questionnaire by World Health Organization measuring Quality of Life by 26 items; SF-12, A 12-item short form to describe mental health; PSS, Perceives stress scale; CSI-3: Couple Satisfaction Index a 3-item questionnaire

HDRS-17 item 14: Genital symptoms: loss of libido.

YMRS item 3: Sexual interest.

FAST item E21: Satisfying sexual life.

Further sociodemographic details included cohabitation status, with 89.2% of partners living with a patient with BD, compared to 58.6% of patients with BD living with a partner. Mean years of education was 16.12 (SD = 1.2) for partners and 15.1 (SD = 3.0) for patients. More partners were employed (72.9%) compared to patients (39.5%), while more patients were unemployed (28.8%) or students (31.5%). The number of children was slightly higher in patients (0.86, SD = 1.2) than in partners (0.69, SD = 1.1) Regarding psychiatric diagnoses, 16.2% of partners reported having a current psychiatric diagnosis, while 17.6% had 1 in the past. Of the patients with BD, 49% had Type 1 BD. Onset age BD was 18 years (Table 2), and illness duration was on average 15.7 years.

Patients with BD had higher HDRS-17 and YMRS scores than their partners (*P* < .001) (Table 2)*,* but none of the 2 groups met the cut-off score for either depression or (hypo)mania. Regarding functioning, patients with BD were rated statistically significant higher on FAST than partners corresponding to mild to moderate functional impairment as compared to no impairment for partners. Both groups rated moderate stress levels (PSS score), with patients with BD reporting more perceived stress than partners (*P* = .022). Partners to patients with BD had impaired mental health and limitations in their everyday life but showed no physical limitation as indicated by SF-12.

## Relationship satisfaction

### Comparison between patients with BD and partners

Looking at relationship satisfaction using the *CSI*-3-total score, there was no statistically significant difference between partners and patients with BD (Table 2). This was consistent in the multiple regression models (Table 3) adjusting for sex and age (*model 1*) and for both sexes, age, *HDRS-17* total, and *YMRS* total scores (*model 2*). In the fully adjusted model (*model 3*), adding *FAST* and *PSS* total scores, there was a significant difference between patients with BD who rated their couple’s satisfaction higher than partners (β = 1.167, 95% CI = 0.000-2.334, *P* = .050) (Table 3). Furthermore, age was statistically significantly associated with relationship satisfaction in all 3 models with higher age meaning less satisfaction (β = -0.104, 95% CI = -0.145 to -0.062, *P* < .001).

**Table 3:** Results of 3 different linear regression models looking at couples’ satisfaction and sexual satisfaction for both patients with BD and partners.

		**Model 1**			**Model 2**			**Model 3**		
		Standardized β	95% CI	*P*-value	Standardized β	95% CI	*P*-value	Standardized β	95% CI	*P*-value
**CSI-3-total**	Group	0.305	-0.636 to 1.245	.523	0.900	-0.203 to 2.003	.109	1.167	0.000-2.334	.050^*^
	Age	-0.111	-0.151 to 0.071	<.001^*^	-0.111	-0.151 to 0.071	<.001^*^	-0.104	-0.145 to 0.062	<.001^*^
	Sex	-0.291	-1.223 to 0.641	.538	-0.176	-1.133 to 0.782	.718	-0.203	-1.162 to 0.756	.676
	HDRS-17				-0.072	-0.156 to .013	.096	-0.045	-0.153 to 0.063	.414
	YMRS				-0.068	-0.191 to 0.055	.274	-0.068	-0.194 to 0.057	.285
	FAST							-0.041	-0.101 to 0.019	.181
	PSS							0.043	-0.052 to 0.137	.374
	R-Square	0.148			0.169			0.179		
**SQOL-02**	Group	-0.013	-0.333 to 0.308	.938	0.212	-0.152 to 0.576	.251	0.401	0.020 to 0.783	.039^*^
	Age	-0.028	-0.042 to 0.014	<.001^*^	-0.028	-0.041 to 0.014	<.001^*^	-0.024	-0.038 to 0.011	.001^*^
	Sex	-0.106	-0.422 to 0.210	.510	0.015	-0.301 to 0.331	.924	-0.018	-0.329 to 0.293	.908
	HDRS-17				-0.050	-0.078 to 0.023	<.001^*^	-0.020	-0.055 to 0.015	.261
	YMRS				0.002	-0.039 to 0.042	.932	0.009	-0.031 to 0.050	.644
	FAST							-0.028	-0.047 to 0.008	.005^*^
	PSS							-0.002	-0.034 to 0.029	.897
	R-Square	0.068			0.123			0.156		

Partners to patients with BD are set as a reference in the category. ^*^Statistically significant *P* ≤ .05.

CSI-3: Couple Satisfaction Index a 3-item questionnaire.

SQOL02: Overall, how would you rate your sex life in the last year? 1. Very bad 2. Bad 3. Neither good 4. Good 6. Very good. HDRS-17: Hamilton

Depression Rating Scale with 17 items version; YMRS, Young Mania Rating Scale; FAST, The Functional Assessment Short Test; PSS: Perceived Stress Scale.

### Comparison with the general population

For comparison, norm scores from a Canadian study^27^ were used. Both partners and patients with BD had lower mean *CSI-3* scores than norm scores (partners = 11.1, BD = 11.6, norm scores = 13.3).

When comparing the 3 *CSI* items included in the present study with the Canadian scores, we found a significant difference (*P* < .050) between the 2 groups and the norm scores, with both patients with BD and partners being less satisfied with their relationship than the Canadian reference group. These scores were not adjusted for age, sex, depressive and manic symptoms, stress, or functioning.

## Sexual satisfaction

### Comparison between patients with BD and partners

*Table 4* provides a detailed overview of the results from the 3 SEXUS questions examining satisfaction, desire, and importance of a good sex life.

**Table 4:** Comparing SEXUS-Questions patients with BD and partners to patients with BD.

**SEXUS-Question**	**Partners**			**Patients with BD**		
	Total (%)	Male (%)	Female (%)	Total (%)	Male (%)	Female (%)
**LYST03: Thinking about the last year, how would you characterize your own and your spouse’s/partner’s desire to have sex?**						
Less sexual desire than partner	26.1	**17.1** ^*^	**39.3** ^*^	29.2	26	32.1
Equal desire for sex	31.9	31.7	32.1	34	30	37.5
Higher sexual desire than partner	42	51.2	28.6	36.8	44	30.4
SQOL01: How important is having a good sex life to you? By sex life, we mean both masturbation and having sex with another person^**^						
Not at all/not very important	20.3	16.7	17.2	6.5	7.8	5.4
Important	24.3	23.8	27.6	29	25.5	32.1
Very/extremely important	55.4	59.5	55.2	64.5	66.7	62.5
**SQOL02: Overall, how would you rate your sex life in the last year?**						
Extremely bad/bad	21.1	19.1	24.1	23.4	27.5	19.6
Neither good nor bad	25.4	26.2	24.1	22.4	17.6	26.8
Good/extremely good	53.5	54.7	51.8	54.2	54.9	53.6

^*^Represents a significant difference within the group between men and women calculated using mean and SD.

^**^Significant difference between partners and patients with BD.

SQOL01 and SQOL02 originally had 5 answer options. Here, the answers are gathered into 3 answer categories for clarity. The original answer options in SQOL01 were: 1. Extremely important 2. Very important 3. Important 4. Not very important 5. Not at all important. In SQOL02 the answer options were 1. Very good 2. Good 3. Neither good nor bad 4. Bad 5. Very bad 6. I have not had a sex life in the last year.

There was no significant difference between partners and patients with BD when rating their sex life in the last year *(SQOL02*). In all 3 regression models, age was a statistically significant variable (model 1 & 2: β = -0.028, 95% CI = -0.042 to -0.014, *P* < .001, model 3: β = -0.024, 95% CI = -0.038 to 0.011, *P* < .001) with higher age associated with less satisfaction with their sexual life in the last year (Table 3). In *model 2,* depressive symptoms showed a significantly negative impact on sexual satisfaction (β = -0.050, 95% CI = -0.078 to -0.023, *P* < .001). In the fully adjusted model, *FAST* had a significant negative impact (*P* = .005). This model showed a statistically significant difference between the 2 groups (β = 0.401, 95% CI = 0.000-2.334, *P* = .039) with patients with BD being more satisfied.

#### Sexual desire and interest

Using the independent *t*-test, partners expressed a non-significant higher sexual desire than patients with BD *(LYST03)*. There was a significant difference between males and females internally in the partner group (*P* = .027). Looking at sexual interest using sexual items from *YMRS* and *HDRS*, we found a significant difference between the patients with BD and partners in the sexual item from *YMRS* where patients with BD had increased sexual interest (*P* = .005). Looking at decreased sexual interest through the sexual item from *HDRS-17*, patients with BD showed a significantly lower interest than partners (*P* = .007) in the original form but no significant difference when moderated into answer options (*P* = .065) (Table 1).

#### Importance of a good sex life

Regarding the importance of a good sex life *(SQOL01),* we found a statistically significant difference between partners and patients with BD (*P* = .006, unadjusted), with patients with BD finding a good sex life more important.

### Comparison to general population

#### Satisfaction with sex life

Both partners and patients with BD were less satisfied with their sex life compared to the age-representative background population (partners = 53.5%, BD = 54.2%, SEXUS: 60.2%). Additionally, a higher percentage of patients with BD and partners rated their sex life the last year as being bad to extremely bad compared to the norm scores from SEXUS (partners = 21.1%, BD = 23.4%, SEXUS = 16%).

#### Desire for sex

We also found that partners to patients with BD wanted sex more often than their partner compared to SEXUS when looking across all age groups (partners = 42%, SEXUS 36%). When comparing results from our study to the specific age group 25-34 years from the SEXUS report, the results were similar when looking at how many had an equal desire for sex (partners = 31.9%, BD = 34.0%, SEXUS = 32.5%). We found that 51.2% of males in the partner cohort wanted sex more often than their partner compared to a male norm score of 56%.

#### Importance of a good sex life

Comparing with norm scores across all age groups from SEXUS, both patients with BD and their partners found it more important to have a good sex life. Compared to the age group 25-34, partners found an excellent sex life less critical, and patients with BD found it equally important (partners = 79.7%, BD = 93.5%, SEXUS = 92.4).

## Correlations

For both partners and patients with BD, there was a statistically significant positive correlation between relationship satisfaction (*CSI*-3-total) and their sex life in the last year (SQOL02) (Table 5). A significant negative correlation was found for both partners and patients with BD when looking at depressive symptoms (*HDRS*-total) and satisfaction with their sex life in the last year.

**Table 5:** Spearman’s correlations between clinical ratings in partners and patients with bipolar disorder (BD).

	**CSI3-total**		**HDRS-total**		**HDRS item 14**		**YMRS-total**		**YMRS-item 3**		**PSS**		**FAST**	
	**PA**	**BD**	**PA**	**BD**	**PA**	**BD**	**PA**	**BD**	**PA**	**BD**	**PA**	**BD**	**PA**	**BD**
**LYST03**														
***P***	.975	.628	.782	.303	.190	.092	.880	.852	.025	.609	.464	.847	.091	.685
***r***	-0.004	-0.048	-0.034	-0.101	-0.160	-0.165	-0.018	-0.018	**0.272** ^*^	0.050	0.090	-0.019	0.205	-0.040
**SQOL01**														
***P***	.331	**.010**	.292	.437	.643	.097	.868	.397	.123	.062	.624	.581	.838	**.022**
***r***	0.117	**0.249** ^*^	-0.127	-0.076	0.056	-0.162	0.020	0.083	0.186	0.182	-0.059	-0.054	0.025	**-0.222** ^*^
**SQOL02**														
***P***	**.000**	**.001**	**.029**	**.016**	.961	**.000**	.785	.789	.171	.422	.245	.163	**.000**	**.009**
***r***	**0.519** ^*^	**0.306**	**-0.259** ^*^	**-0.233** ^*^	0.006	**-0.398** ^*^	-0.033	-0.026	-0.166	0.079	-0.140	-0.136	**-0.425** ^*^	**-0.251** ^*^
**CSI3**														
***P***			**.027**	.430	.083	.571	.253	.655	.384	.885	.229	.769	**.004**	-.164
***r***			**-0.257** ^*^	-0.078	-0.203	-0.056	-0.135	-0.044	0.103	0.014	-0.141	0.029	**-0.335** ^*^	0.095
**PSS**														
***P***	.229	.769	.342	**.000**	.233	**.008**	.059	.098	.888	.573			.337	**.000**
***r***	-0.141	0.029	0.112	**0.546** ^*^	0.140	**0.253** ^*^	0.220	0.158	-0.017	-0.054			0.113	**0.522** ^*^
**FAST**														
***P***	**.004**	.095	**.000**	**.000**	**.003**	**.000**	**.001**	.533	**.040**	.058	.337	**.000**		
***r***	**-0.335**	-0.164	**0.624** ^*^	**0.569** ^*^	**0.336** ^*^	**0.333** ^*^	**0.391** ^*^	0.060	**0.242** ^*^	-0.182	0.113	**0.522** ^*^		

^*^Statistically significant *P* ≤ .050.

PA, partners, BD, patients with bipolar disorder.

HDRS-17, Hamilton Depression Rating Scale with 17 items version; YMRS: Young Mania Rating Scale; FAST, The Functional Assessment Short Test; PSS, Perceived Stress

scale; CSI3, Couple Satisfaction Index a 3-item questionnaire

HDRS-D item 14: Genital symptoms: like loss of libido and menstrual disturbances.

LYST03: Thinking about the last year, how would you characterize your own and your spouse’s/partner’s desire to have sex? SQOL01: How important is having a good sex life to you? By sex life, we mean both masturbation and having sex with another person.

For both groups, a statistically significant negative correlation was observed between total *FAST* scores and ratings of their sex life during the last year. Hence, better overall functioning was correlated with a more positive rating.

## Discussion

In this study, we identified differences in relationship and sexual satisfaction between 111 patients with BD and 74 partners to patients with BD. Patients with BD were more satisfied with their relationship, reported greater satisfaction with their sexual life, and valued a good sexual life more compared to partners. Patients also exhibited both decreased and increased sexual desire. Compared to norm scores, patients with BD and partners showed less satisfaction with both relationship and sexual life.

Being a close relative to an individual with BD can be demanding and lead to caregiver burden, as the need of the partner conflicts with the needs of the individual with BD^21^ and is negatively correlated with marital functioning and adjustment.^39^ This burden aligns with the present findings that partners experience lower levels of relationship satisfaction and more perceived stress. It has been shown that partners experience distress and caregiver burden, even when their partners with BD are in euthymic periods, as they worry about recurrence of a new episode.^40^ Furthermore, patients with BD’s mental health are adversely affected by high levels of caregiver burden, which underlines the importance of focusing on the partners in treatment.^20^ Regarding caregiver burden, it is important to consider the mental health disorders of partners as there may contribute to heightened caregiver burden, leading to increased stress levels associated with managing one’s own mental health condition.

In the literature, it is described that partners to patients with BD have more psychiatric symptoms than individuals with a healthy partner.^19^ We observed that more partners had a mental health disorder than the average population, as 14.8% of partners stated that they had a current depression diagnosed by either a physician or psychologist. However, only 4.1% of partners were clinically considered to have depression according to their rating on the *HDRS-17* scale (score ≤ 13). The phenomenon of assortative mating should also be considered, as previous studies have shown that patients with psychiatric disorders more often find a partner who also has a psychiatric disorder.^41^^,^^42^ It is hence essential to focus on partners to patients with BD in treatment and improve mental health in this group as this amends the quality of life for partners^43^ and seems to be a protective factor for the individual with BD.^8^^,^^10^

Both patients with BD and partners reported moderate perceived stress levels. This finding could be due to caregiver burden, which could explain the partners’ increased stress levels. Other factors, such as physical disease, could also be an explanation. When looking at partners’ quality of life (*SF-12,*  Table 2), they only rated lower than the reference score in their mental score and not in their physical score, indicating that the increased perceived stress was not due to physical impairment.

Additionally, the sample included in the Canadian study using the Couple Satisfaction Inventory was approximately 10 years older than our 2 study samples, potentially impacting the outcomes. As we found age to be negatively correlated with relationship satisfaction, patients with BD and partners may be even less satisfied with their relationships if the norm scores had been from a younger population. A systematic review from 2021 supports this possibility as it showed that relationship satisfaction declined from age 20 to 40, reaching a low point and hereafter increasing again reaching a plateau around age 65.^43^

Our primary outcome regarding sexual satisfaction was the *SEXUS* question on sexual satisfaction the last year (SQOL02). Supplementary to this, we included 2 other *SEXUS* items and the sexual items from *HDRS-17*, *YMRS*, and *FAST*. These items covered other factors such as sexual desire and the importance of a good sex life and was included as previous studies show that a discrepancy in these matters led to less sexual satisfaction to cover the complex field thoroughly.^44^^,^^45^ Partners were less satisfied with their sexual lives compared to patients with BD. To our knowledge, no studies have compared partners and patients with BD on this aspect before. Both groups were less satisfied with their sexual life when compared to age-matched norm scores from project SEXUS. It must be noted that the SEXUS cohort consisted of individuals with various types of civil status, with only 50% being in a current relationship, which could have impacted their answers. However, our findings are consistent with previous findings where both patients with BD and partners experience more sexual dissatisfaction.^12^^,^^13^^,^^18^ For both groups, higher age was negatively correlated with sexual satisfaction, aligned with norm scores.^3^^,^^28^ Finally, we found a positive correlation between overall sexual satisfaction (SQOL02) and relationship satisfaction (CSI-3-total) (Table 5) that supports our assumption about the relation between a satisfying sex life and a good relationship. Here, the partners wanted sex more often than both patients with BD and the average population, which could be explained by having more males in the partner group. Indeed, post-analyses revealed a difference between men and women; females reported that they wanted less sex than males. This finding aligns with previous research and norm scores,^46^ though some studies suggest that the actual sex difference may be smaller, as cultural norms can cause women to downplay their sexual desire and men to exaggerate theirs.^47^

Looking at sexual desire using sexual items from *HDRS*-17 and *YMRS*, patients with BD had lower sexual interest and higher sexual interest than their partners, respectively, which is consistent with previous research.^13^ Interestingly, other studies have indicated that bipolar depression could be differentiated from unipolar depression by lacking decreased sexual interest during depression. For instance, a chart review found that hospitalized adolescents having bipolar depression had an increased sexual interest based on rating the sexual item from *YMRS* compared to adolescents with unipolar depression.^48^ This finding was supported further in a qualitative study where 2 male participants described having a destructive sex drive and not reduced sex drive when being depressed.^49^ However, these findings are indicative and yet need further investigation. Nonetheless, 33% of patients with BD were clinically considered mildly depressive, which could affect both the higher and lower sexual interest measured in patients with BD. Examining the sexual item in YMRS, 20.8% of patients with BD showed increased sexual interest but only 3.6% were clinically considered mildly manic. Finally, the patients with BD answered the questionnaires more polarized—either extremely good or extremely bad—compared to partners and norm scores. A finding that the nature of the disorder could explain.

### Limitations and strengths

A limitation was the cross-sectional design. Our norm scores for CSI^27^ were limited as these did not use national norm scores. However, the norm scores were from a Western country, minimizing cultural differences. A limitation of this study is that we only had access to the published results from the included studies from the general population,^3^^,^^27^^,^^28^ not the original raw data. This restricted our ability to conduct further statistical analyses or explore additional variables beyond those presented. Access to the original data could have provided opportunities for more in-depth analysis, which we consider a potential direction for future research. Furthermore, CSI-3-total is not a validated questionnaire, but it was used due to a lack of data on the fourth item in the partner cohort and, therefore, for comparison reasons, was converted into a total of the first 3 questions (CSI-3-total). Regarding sexual satisfaction, information on physical illness could be of interest as it can impact one’s sexual functioning and thereby satisfaction. However, as our 2 cohorts are young, this will unlikely influence the results. Another limitation of this study is that our cohort consists of patients with BD who are generally less severe, as they are not hospitalized and relatively newly diagnosed. Additionally, their overall functioning is relatively good, as indicated by their FAST scores. These factors may limit the generalizability of our findings to individuals with more severe forms of BD. Lastly, we also aimed to examine matched patients with BD and their respective partners, and we had a subsample consisting of 20 couples. The subsample was relatively small and limited because the couples were not rated at the same time, and we have chosen not to run further analyses on this subsample.

### Implications

In future studies, it is suggested to include matched couples to further explore correlations between the affective periods expressed in patients with BD and their partners’ corresponding satisfaction with both relationship and sexual life. It is further of interest to investigate the high perceived stress levels and higher prevalence of psychiatric disorders reported by partners and to what extent this is related to having a partner with BD, eg, assortative mating.^41^ Finally, examining the caregiver burden in partners to patients with BD could add new angles on which aspects of caregiver burden affect relationship satisfaction to make a targeted effort to enhance relationship satisfaction.

## Conclusion

Patients with BD valued a good sex life more and were more satisfied with their sexual lives and showed increased sexual interest compared to partners. Compared to the average population, both patients and partners to patients with BD showed less satisfaction with their relationship and sexual satisfaction. Both partners and patients reported moderate perceived stress levels, and more partners compared to the general population had a psychiatric diagnosis. These findings emphasize the clinical relevance to further study whether there is a need for specific interventions that address relationship and sexual satisfaction disparities in couples affected by BD.
